# Cognitive Fatigue, Sleep and Cortical Activity in Multiple Sclerosis Disease. A Behavioral, Polysomnographic and Functional Near-Infrared Spectroscopy Investigation

**DOI:** 10.3389/fnhum.2018.00378

**Published:** 2018-09-20

**Authors:** Guillermo Borragán, Médhi Gilson, Anne Atas, Hichem Slama, Andreas Lysandropoulos, Melanie De Schepper, Philippe Peigneux

**Affiliations:** ^1^Neuropsychology and Functional Neuroimaging Research Unit, Université Libre de Bruxelles, Brussels, Belgium; ^2^Centre de Recherches en Cognition et Neurosciences and ULB Neurosciences Institute, Université Libre de Bruxelles, Brussels, Belgium; ^3^Consciousness, Cognition and Computational Group, Université Libre de Bruxelles, Brussels, Belgium; ^4^Cognitive Neurosciences Research Unit, Université Libre de Bruxelles, Brussels, Belgium; ^5^Department of Clinical and Cognitive Neuropsychology, Erasme Hospital, Université Libre de Bruxelles, Brussels, Belgium; ^6^Neuroimmunology Unit – Multiple Sclerosis, Erasme Hospital, Université Libre de Bruxelles, Brussels, Belgium

**Keywords:** cognitive fatigue, sleep, cortical activity, multiple sclerosis, fNRS

## Abstract

Patients with multiple sclerosis (MS) disease frequently experience fatigue as their most debilitating symptom. Fatigue in MS partially refers to a cognitive component, cognitive fatigue (CF), characterized by a faster and stronger than usual development of the subjective feeling of exhaustion that follows sustained cognitive demands. The feeling of CF might result from supplementary task-related brain activity following MS-related demyelination and neurodegeneration. Besides, CF in MS disease might also stem from disrupted sleep. The present study investigated the association between the triggering of CF, task-related brain activity and sleep features. In a counterbalance mixed design, 10 patients with MS and 11 healthy controls were exposed twice for 16 min to a CF-inducing dual working memory updating task (TloadDback) under low or high cognitive demands conditions, counterbalanced. Considering known inter-individual differences and potential cognitive deficits in MS, the maximal cognitive load of the task was individually adapted to each participant’s own upper limits. During the experimental sessions, cortical brain activity was measured using near-infrared spectroscopy (NIRS) during the CF-induction task, and in a resting state immediately before and after. Ambulatory polysomnography recordings were obtained on the nights preceding experimental sessions. When cognitive load was individually adapted to their processing capabilities, patients with MS exhibited similar than healthy controls levels of subjectively perceived CF, evolution of performance during the task, and brain activity patterns. Linear mixed models indicate a negative association between oxygenation level changes in the dorsolateral prefrontal cortex (DLPFC) and the triggering of subjective CF in patients with MS only. Longer total sleep time was also associated with higher CF in MS patients. These results suggest that controlling for cognitive load between individuals with and without MS results in a similar task-related development of subjective CF. Besides comparable performance and cortical brain activity between groups, mixed model analyses suggest a possible association between CF, DLPFC activity and sleep duration in MS disease.

## Introduction

Multiple sclerosis (MS) is a neurodegenerative disease characterized by inflammation, demyelination and axonal degeneration, often accompanied by motor and cognitive deficits (Trapp et al., 1998). MS is the first cause of disability in young populations in modern societies (Rosati, 2001), with high social care costs (Adelman et al., 2013). Fatigue is the most common symptom, reported by up to 90% of patients with MS (Walker et al., 2012). Moreover, fatigue is for more than half of the patients reported as the main cause of interference with daily living activities and quality of life (Comi et al., 2001). Multifactorial in nature, the phenomenon can be classified into primary fatigue, if the causal mechanisms are directly related to the disease, and secondary fatigue, if the mechanisms are not specific to the disease (Kos et al., 2008). Primary fatigue has been associated with brain atrophy (Calabrese et al., 2010; Genova et al., 2013), functional brain connectivity changes (Cader et al., 2006; Cruz Gómez et al., 2013), disease severity (Pittion-Vouyovitch et al., 2006) as usually assessed with the Expanded Disability Status Scale (EDSS; Kurtzke, 1983), dopamine imbalance (Dobryakova et al., 2015), neuroendocrine level variations (e.g., pro-inflammatory cytokines; Heesen et al., 2006) and compensatory brain overactivation patterns (Kos et al., 2008; Leocani et al., 2008). For its part, secondary fatigue has been associated with factors such as mood and personality (Bol et al., 2009), depression (Strober and Arnett, 2005) or sleep disturbances (Veauthier and Paul, 2014). In spite of the large number of variables associated with fatigue, the symptom is still barely understood, and pharmacological treatments are barely efficient (Bol et al., 2009). One potential explanation for inconclusive results might stem from a lack of specification of the term “fatigue.” Indeed, the physical, cognitive and psychosocial aspects of fatigue are often intermixed during the assessment of the symptoms. For instance, these different aspects are not explicitly segregated in the most popular fatigue severity scale inventory (FSS; Krupp et al., 1989). Physical and cognitive fatigue (CF) are better distinguished in the fatigue scale for motor and cognitive functions (FSMC; Penner et al., 2009), but fatigue is only assessed as a trait in this scale. However, patients with MS experience variations in their fatigue state in daily life, and might be especially vulnerable to CF triggered online by task-related cognitive demands. Although several studies might support the assumption that CF develops faster in patients with MS than in controls (Lehmann et al., 2012; Genova et al., 2013; Sandry et al., 2014), it is worth noticing that in these studies, all participants were exposed to the same task conditions, which might bias the evaluation of CF. Indeed, since attentional (Urbanek et al., 2010) and working memory (Brissart et al., 2012) deficits are often reported in MS, cognitive demands for the same task conditions are likely to be much higher for patients than controls, thus eventually leading to higher pressure and CF levels. Therefore, to delineate the development of task-related CF in MS disease from other factors, task demands should be carefully adapted to each patient’s individual cognitive processing capacity.

As mentioned above, compensatory cortical overactivity needed to maintain performance at normal levels in visual (Werring et al., 2000; Colorado et al., 2012), motor (Pantano, 2002; Tomassini et al., 2012) and cognitive (Forn et al., 2006; Audoin et al., 2008; Colorado et al., 2012) tasks is viewed as a potential cause for CF in MS disease. Besides, it was proposed that compensatory brain overactivity required to cope with the neurodegenerative process in MS is a cause of acute fatigue (Leocani et al., 2008). However, evidence remains scarce (DeLuca et al., 2008; Genova et al., 2013) and no predictors of this capacity have been described beyond cognitive reserve (Bonnet et al., 2010; Das et al., 2014). Additionally, sleep disturbances have been reported in MS, and especially fragmented sleep (Kaminska et al., 2011). Sleep is involved in brain plasticity maintenance (Dang-Vu et al., 2006) and is hypothesized to support the cellular homeostasis restorative processes allowing the brain to cope with daily cognitive challenges (Tononi and Cirelli, 2014). Thus, sleep fragmentation in MS might reduce the efficacy of brain compensatory mechanisms to maintain performance, eventually resulting in a faster triggering of CF. If this relation is proven true, then sleep medicine interventions in the clinical management might contribute to improve quality of life in MS patients.

In this framework, we aimed at investigating the putative triangular relationship between the triggering of CF, cortical overactivity patterns and sleep disruptions. To assess CF unbiased by interindividual differences face to the same task conditions, we used a paradigm in which task-related cognitive load can be individually adapted to each participant’s maximal performance level (TloadDback task; Borragán et al., 2017). Participants were then administered in a counterbalanced design the same task on two separate occasions, once in a high cognitive load (HCL) and once in a low cognitive load (LCL) condition. Cortical activity was recorded before, during and after the CF-inducing task (HCL and LCL on two separate days) using functional near infrared spectroscopy (fNIRS). Polysomnography recordings were obtained for the nights preceding each of the experimental days.

## Materials and Methods

### Participants

Ten patients with relapsing-remitting MS (RRMS) according to the criteria of McDonald et al. (2001) and 11 healthy controls were included in the present study. Patients were recruited over a 12-month period by an experienced neurologist (AL), and were in remission from the time of the clinical examination until the NIRS imaging session. All participants gave their written informed consent to participate in the present study conducted in agreement with the Declaration of Helsinki, and approved by the institutional ethics committee of the ULB-Erasme Hospital. MS and control groups had similar level of education (in years) but were not matched for age (**Table 1**). Therefore, age was included as a confounding covariate in the statistical analyses. Demographic and neuropsychological data are reported in **Table 1**. Due to a data transfer problem, fNIRS data were not accessible for two patients. Likewise, PSG was not obtained in one MS patient and one control participant, due to bad quality in the EEG signal.

**Table 1:** Demographic and neuropsychological data in healthy controls and patients with MS disease.

		Healthy controls (*n* = 11)	Patients with MS (*n* = 10)	p/BF	p/(age as covariate)/BF
Age		34.18 ± 4.64	42.1 ± 7.1	**<0.05/>7^∗^**	–
Disease duration (years)		–	7.6 ± 5.9	–	–
Last manifestation (years)		–	3.44 ± 3.4	–	–
EDSS (range)		–	1.65 ± 0.77 (0-3)	–	–
Years of education		15.64 ± 1.9	14.1 ± 2	>0.081/1.2	>0.16/0.6
Depression		3 ± 2.7	5.2 ± 3.2	>0.1/1.1	<0.1/1.2
Anxiety		4.7 ± 2.1	7.1 ± 2.6	**<0.05^∗^**/2.2	>0.18/1.6
sleep	/PSQI	4.2 ± 1.7	7.6 ± 3.2	**<0.01/4^∗^**	**<0.05/>3^∗^**
	/H&S	49.3 ± 8.6	55.9 ± 12.5	>0.17/0.8	>0.79 /0.6
FSMC	/ cognitive	22 4 ± 6 8	32.1 ± 7	**<0.005/9^∗^**	**<0.05/12^∗^**
	/ physical	21 2 ± 8.2	36 ± 4.6	**<0.001/252^∗^**	**<0.001/170^∗^**
	/ psychosocial	4.7 ± 1.8	6.6 ± 1.8	**<0.05^∗^**/2.4	>0.49/1.2
Digit Span^direct^		70 ± 3%	54 ± 17%	**<0.05/>3^∗^**	>0.24/1
Digit Span^inverse^		54 ± 18%	46 ± 15%	>0.23/0.6	>0.31/0.6
Visual Span^direct^		76 ± 4%	64 ± 11%	**<0.05^∗^/**2.4	**<0.05^∗^/**2.3
Visual Span^inverse^		73 ± 20%	61 ± 12%	**<0.05^∗^/**2.5	**<0.05^∗^/**2.7
TMT_Numbers Sequencing_		22 ± 2.7 s	27 ± 10 s	**<0.05^∗^/**2.3	>0.15/1.7
TMT_Switching_		51 ± 23 s	66 ± 40 s	**<0.05/>3^∗^**	<0.06/2.8
Stroop Inhibition		85 ± 14 s	110 ± 18 s	**<0.005/13^∗^**	**<0.005/17^∗^**
SDMT^a^		66 ± 15	53 ± 6	**<0.05/>3^∗^**	**<0.05/8^∗^**
PASAT-3s^b^		46 ± 8	38 ± 12	=0.09/1.3	**<0.005^∗^/**14
PASAT-2s^b^		36 ± 11	25 ± 11	**<0.05^∗^/**2.9	**<.005/3.9^∗^**

*Values are mean and standard deviation (m ± std). P-values, computed using non-parametric Mann–Whitney tests/Bayes factors (BF) are provided on the 3rd and 4th columns. BFs < 0.33 suggest equality between H0 and H1 and BFs > 3 inequality. Values in between [0.33 – 3] denote inconclusive results. EDDS, Expanded Disability Status Scale (Kurtzke, 1983), an universal scale to rate neurologic impairment in multiple sclerosis. ^a^In number of identified and written elements. ^b^Number of correct responses. ^∗^New ANCOVA p-values with age as covariate are displayed on right column. Statistically significant values are highlighted in bold font.*

### Experimental Design and Procedure

**FIGURE 1:**
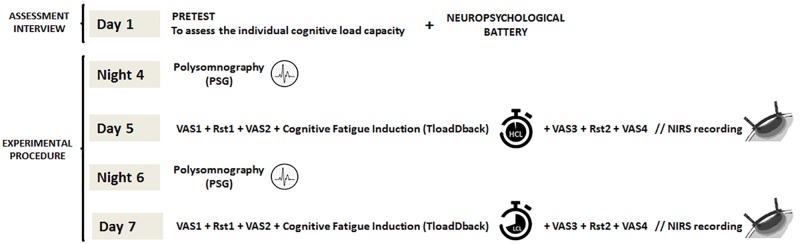
Experimental design (overview). On Day 1, participants’ maximal cognitive load is calibrated on the TloadDback task (pretest), followed by a neuropsychological assessment. On the nights of Day 4 and Day 6, nocturnal sleep is recorded at home using ambulatory PSG. On Day 5 and Day 7 after the PSG night, participants are administered the CF-inducing TloadDback task under either the high (HCL) or low (LCL) cognitive load condition, counterbalanced. Evolution of subjective feelings of CF and sleepiness were assessed throughout the testing session using visual analog scales (VAS). Cortical brain activity was recorded using fNIRS both during the TloadDback task and during the resting state sessions immediately before and after the task.

The experiment was carried out over 7 consecutive days (**Figure 1**). On Day 1, a Pretest session (35–40 min duration) aimed at determining for each participant separately her/his maximal cognitive load capacity on the CF-inducing TloadDback task (see below). I.e., maximal load was defined as the fastest stimulus presentation rate at which the participant was still able to maintain an accuracy performance > 85%. This fastest presentation rate defined the HCL condition. For the LCL condition, stimulus presentation rate was slowed down by 50%, making it easier to process the ongoing stimuli. This procedure was shown efficient to induce high vs. low levels of CF in healthy participants (Borragán et al., 2016, 2017). Following the Pretest session at Day 1 and after a brief rest period (∼ 10 min), a neuropsychological battery test (average duration 45 min) was administered. On Day 5 and Day 7, participants were first equipped with the fNIRS optodes (20–25 min), and then administered during 16 min the TloadDback task under either the high (HCL) or the low (LCL) cognitive load condition, in a counterbalanced order. Cortical activity was recorded using fNIRS during the 16 min of TloadDback task practice, and during two 4-min resting state (Rst1) sessions, immediately before and after the TloadDback task. In the resting state sessions, participants were asked to look at a fixation cross on a computer screen. Before and after each resting state and the TloadDback task (i.e., at 4 time points per testing day, see **Figure 1**), participants were asked to report their subjectively perceived levels of CF and sleepiness using visual analog scales Thus, a whole testing session lasted approximately for 50 min. The night before each testing session, sleep was recorded using ambulatory polysomnography at the participant’s home. Polysomnography was set-up and configured by the principal investigator (GB) 1–2 h before participants’ usual bedtime. To prevent circadian confounds, all testing sessions took place at the same time of day within each participant, either in the morning (9–12 h) or in the afternoon (14–19 h).

### Tasks and Material

#### Cognitive Fatigue Induction Paradigm (TloadDback)

Cognitive fatigue was induced using the TloadDback task (for details see Borragán et al., 2017), a dual task that combines a working memory updating task (N-back; Kirchner, 1958) and a parity number decision task. In this task, digits and letters are displayed on screen in alternation. Participants are instructed to press the space bar with their left hand every time the displayed letter is the same than the penultimate letter, or to indicate whether the displayed digit is odd or even by pressing “1” or “2” on the numeric keypad. Combining two tasks featuring different processing requirements ensures a large recruitment of working memory resources, eventually leading to a decrease in performance and the intensification of the subjective feeling of mental exhaustion (Borragán et al., 2017). The task includes a separate pretest session (on Day 1) during which the individual’s maximal cognitive load capacity is determined as the shortest duration the stimuli can be presented (stimulus time duration; STD) for a performance still > 85% accuracy. During this pre-test session, STD was initially set at 1500 ms for the first block (30 letters and 30 digits, alternating), then speeded up by 100 ms for the next block if accuracy performance was >85%. The procedure was repeated until accuracy performance dropped < 85%, indicating that the participant’s cognitive load limit had been reached. STD was then slowed down by 100 ms, and again speeded up by 100 ms in case of success, to improve the reliability in the pre-test. After three failures to reach accuracy > 85% at a given STD, the immediately preceding STD at which performance was still successful was determined as the individual’s maximal capacity, which was used in the testing session to define the HCL condition. For the LCL condition, STD was made longer by 50%, eventually ensuring comparable levels of low vs. high cognitive demands between individuals. CF was assessed subjectively using a Visual Analog Scale for fatigue (VASf: Lee et al., 1991), and objectively by computing the evolution of performance (accuracy) across four 4-min quartiles (i.e., t1, t2, t3, and t4) during the 16 min duration of the TloadDback task. Additionally, self-reported sleepiness was assessed using a VAS scale (VASs).

#### Neuropsychological Battery

Executive function and information processing capacities were evaluated using several neuropsychological tests (French versions). The digit and visual spans of the Wechsler Adult Intelligence Scale (WAIS; Wechsler, 1981) consist on a verbal repetition of numbers (Digit) or spatial positions (Visual) in direct and reverse order; they are usually employed to assess short-term and working memory functioning. The Trail-Making Test (Reitan, 1958) mostly tests mental flexibility. It features 25 circles distributed over a sheet. In the Numbers Sequencing condition, the circles are numbered 1 – 25 and the individuals must draw lines to connect the numbers in ascending order. In the Switching Part condition, the circles include both numbers (1 – 13) and letters (A – L) and the individuals must connect the circles alternating numbers and letters in ascending order again (i.e., 1-A-2-B-3-C, etc.). Successful TMT performance requires adequate letter and number recognition, mental flexibility, visual scanning and motor function (Gaudino et al., 1995). In the Stroop task (Stroop, 1935), participants are asked to provide the ink color of a written color name. Interference due to automatic reading makes providing the ink color more difficult and slower when the written color name is different from the printed color. The test thus assess the inhibitory component of selective attention and processing speed ability (Lamers et al., 2010). The written section of the Symbol Digit Modalities Test (SDMT; Smith, 1968) requires individuals to identify nine different symbols corresponding to the numbers 1 through 9 and to manually fill the blank space under each symbol with the corresponding number, as fast as possible. The final score is computed as the number of correct symbols identified and written down in a 90 s interval. The neuropsychological assessment ended up with the Paced Auditory Serial Addition Test (PASAT; Gronwall, 1977). In this test, single digits are presented either every 3 (trial 1) or 2 (trial 2) seconds, and individuals must add each new digit to the one immediately prior to it. Final score is the total number of correct sums given (out of 60 possible) in each trial. The test is widely employed in MS disease to evaluate processing speed, flexibility and calculation ability (Rudick et al., 1997). To moderate the cognitive workload caused by neuropsychological assessment, tests were administered in the order in which they are described in the text.

#### Polysomnography

To investigate sleep characteristics in the nights preceding the behavioral and fNIRS imaging sessions, we used ambulatory polysomnography (BrainRT EEG/PSG system, OSG BVBA, Belgium). PSG recording included standard electroencephalogram (EEG) at locations Fp1, Fp2, F3, F4, C3, C4, P3 and P4 according to the standard 10–20 system, electrooculogram (EOG), electromyogram (EMG), and electrocardiogram (ECG). Respiratory events were detected using abdominal and thoracic belts, oral/nasal airflow with a nasal pressure transducer, an oronasal thermocouple and oxygen saturation levels measured by finger pulse oximetry. PSG scoring was performed in agreement with the American Academy of Sleep Medicine (AASM) criteria (Iber et al., 2007). Each 30-s epoch was visually classified by a qualified expert somnologist (MG) as Wake, N1, N2, N3 or REM sleep stage. Sleep onset was defined as the first 30-s epoch scored N2, SWS or REM and final awakening as the last 30-s epoch scored N2, SWS or REM. Sleep period time was defined as the time interval separating sleep onset from final awakening. Total sleep time was calculated as the sleep period time minus the duration of intra-sleep awakenings, and sleep efficiency was expressed as the percentage of total sleep time during the sleep period time. The apnoea-hypopnea index (AHI) was computed in all participants. The cut-off score for the presence of apnoea was AHI > 5 events per hour. Micro arousals were computed as awakening periods > 3 s and <15 s. EEG data were visualized using the PRANA software package (PhiTools, Strasbourg, France).

#### fNIRS Acquisition

**FIGURE 2:**
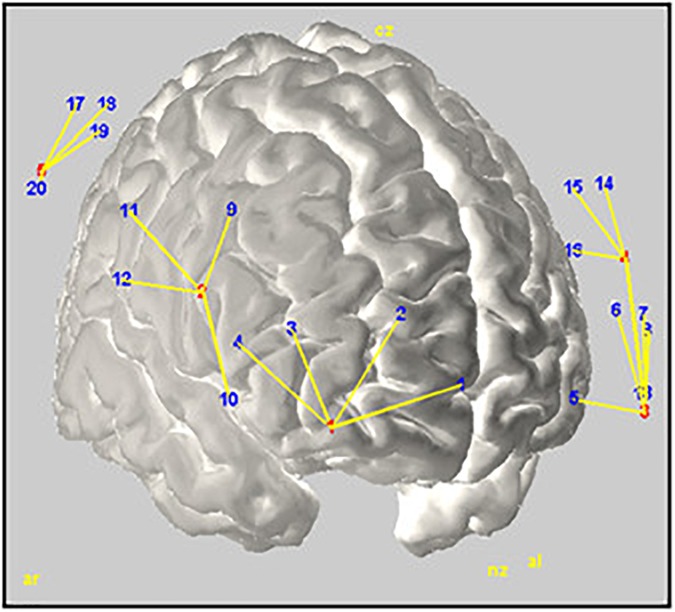
Rendering of the estimated optodes positions on a MNI template brain. Red and blue numbers represent sources and detectors, respectively; yellow lines illustrate their interconnection. The figure was created using Homer2-AtlasViewer (Aasted et al., 2015).

Variations in haemodynamic cortical activity were recorded using a multichannel fNIRS system (BrainSight, V2.3b16, NIRS, Rogue Research Inc., Canada) with two continuous wavelengths (685 and 830 nm). The set-up featured a total of 24 channels (detectors) created around 6 source optodes encompassing 3 areas in each cerebral hemisphere: ventrolateral prefrontal cortex (VPFC), dorsolateral prefrontal cortex (DLPFC), and Inferior Parietal Cortex (IPC). Channels in each area were averaged to provide a general measure of brain activity. Optodes were positioned and located using a 3-D coordinates system combined with a Polaris localization device (see **Figure 2**). Channels displacements during the experiment were minimized using elastic tape. Detector optodes were located at an approximate distance of 3 cm from the source optodes. Functional NIRS signals were recorded at a sample rate of 20 Hz. To limit the natural trend appearing within signal recording, separate recordings were made for the Rst1, TloadDback and Rst2 periods. This made also easier to remind the TloadDback task instructions. Thus, there is a short temporal gap between the 3 successive recordings conducted within one individual.

### fNIRS Analysis

Oxyhemoglobin ΔHbO and deoxyhemoglobin ΔHbR were computed at each time point for each channel using the modified Beer–Lambert law. For each participant, raw recorded absorption units were normalized then low-pass filtered (0.009 – 0.08 Hz) to attenuate high-frequency noises arising from Mayor waves: respiration, cardiac pulsations and optodes movements. Furthermore, to improve signal quality, noisy channels were automatically detected and removed from the analysis using an automated algorithm based on correlation analysis (Guerrero-Mosquera et al., 2016). In a nutshell, data preprocessing was performed as follows. First, data were located using digital triggers, then a first normalization of AUs (Absorption Unities) was performed followed by the band pass filtering implemented in Homer2 (Huppert et al., 2009). Then, a new normalization was performed to allow computing new analysis coming from a corrected baseline output. This normalization was done with regard to the individual data points. Finally, the concentration of the different chromophores was computed using Homer2’s code *hmrOD2Conc.*

The fNIRS signal was analyzed computing two different indicators of brain activity. First, cortical activity changes during practice of the TloadDback task and during resting states were estimated computing Cerebral Oxygen Exchange (COE; see e.g., Sano et al., 2013; Yoshino et al., 2013), an indirect measure of brain metabolism computed as the difference between HbR and HbO. Higher COE indicates reduced oxygenation levels, and vice-versa. Task-related changes in brain activity during the ensuing resting state period (Rst) were estimated by computing the amplitude of low frequency fluctuations (ALFF; Yu-Feng et al., 2007) of the total hemoglobin concentration. ALFF is viewed as a good measure of spontaneous neural activity, appropriate to detect state-dependent resting brain changes associated with CF (Gui et al., 2015).

### Statistics

Statistical analyses were computed following Fritz et al. (2012) recommendations. Mean (m) ± Standard Deviation (std) are reported as measures of central tendency, and size effects are reported as partial eta squares (η^2^). Mean squared errors (MSE) are included in the ANOVAs. Significance level was set at *p* < 0.05 (two-tailed) and Tukey HSD test was employed for *post hoc* corrections. Bayesian analyses were conducted to estimate the likelihood of the null hypothesis.

## Results

Demographic parameters and results on neuropsychological tests are provided **Table 1**. MS patients were significantly older than controls (*p* < 0.05, Bayes factor [BF] > 3). For this reason, age was considered as a covariate in the subsequent analyses. When considering the covariate age, sleep quality as measured through the PSQI was lower (*p* < 0.05, *BF* > 3) and levels of cognitive and physical fatigue higher (*p* < 0.05, *BFs* > 3) in MS patients compared to healthy controls. Besides, patients’ performance was lower in both direct and indirect visual span tests (*p* < 0.05, *BFs* = inconclusive), the Stroop task (*p* < 0.01, *BF* > 17), the SDMT (*p* < 0.05, *BF* > 8) and the PASAT-3s (*p* < 0.01, *BF* = inconclusive) and PASAT-2s (*p* < 0.01, *BFs* > 3).

### Behavioral Data

#### Pretest

First, we investigated between-group differences in the optimal stimulus time duration (STD) needed to maintain successful performance (accuracy > 85%) in the TloadDback task at Pretest on Day 1. An ANCOVA performed on the STD with a between-subject factor Group and the confound covariate Age disclosed significant differences [*F*_(1,18)__=_ 5.78; *p* < 0.05; *MSE* = 0.23; ${\text{η}}_{\text{p}}^{2}$ = 0.24]. Participants in the control group had a shorter (faster) STD (average ± standard deviation 811 ± 0.094 ms) than participants with MS (960 ± 126 ms).

### Self-Reported Evolution of Cognitive Fatigue and Sleepiness (VAS)

**FIGURE 3:**
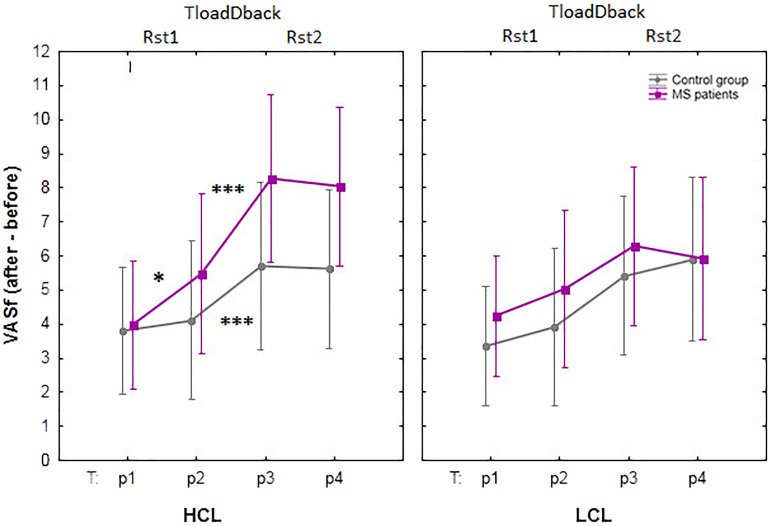
Evolution of self-reported cognitive fatigue (CF) using visual analog scales for fatigue (VASf). HCL: high cognitive condition; LCL: low cognitive condition; Rst: pre- (Rst1) and post- (Rst2) task resting state sessions (5 min); TloadDback: CF-inducing dual task (16 min). The interaction between Condition (HCL vs. LCL), Period (pre-Rst1 p1, pre-TloadDback p2, post-TloadDback p3, post-Rst2 p4) and Group (Healthy controls vs. MS patients) was significant (*p* < 0.05). Asterisks indicate significant *p*-values after Tukey *post hoc* correction: ^∗^*p* < 0.05; ^∗∗^*p* < 0.01, and ^∗∗∗^*p* < 0.001. *n.s*, non-significant.

Subjective evolution of CF was assessed using the VASf before (p1) and after (p2) the resting state session Rst1, after the TloadDback task (p3) and after resting session Rst2 (p4), see **Figure 3**. A mixed repeated-measure ANCOVA was computed on VASf scores with within-subject factors Condition (HCL vs. LCL) and Period (p1 vs. p2 vs. p3 vs. p4), between-subjects factor Group (Healthy controls vs. MS patients) and confounding covariate Age. Results disclosed a triple interaction Condition × Period × Group [*F*_(3.51)__=_ 3.56; *p* < 0.05; *MSE* = 0.97; ${\text{η}}_{\text{p}}^{2}$ = 0.17; BF was < 0.33]. Tukey *post hoc* tests showed that CF similarly increased in both groups in the LCL condition (*all p* < 0.88) with significant differences found between p1- p3 and p1- p4 for both groups (*all p* < 0.05), i.e., from before the pre-task resting state session (p1) to after the TloadDback (p3) and after the post-task resting state session (p4). CF was not significantly modified from immediately before (p2) to immediately after (p3) the TloadDback task in the LCL condition (*all p* > 0.16). In the HCL condition, CF increased in both groups (*all p* < 0.54) with significant differences found again between p1 – p3 and p1 – p4, but also from immediately before the task to immediately after (p2 – p3) and to after the second resting state session (p2 – p4), in both groups (*all p* < 0.05). Finally, CF significantly increased in MS patients from the pre- (p1) to the post- (p2) resting state session Rst1 (*p* < 0.05), i.e., before exposure to the CF-inducing TloadDback task. This effect was not present in healthy controls (*p* > 0.9).

Similar analyses computed on sleepiness scores failed to disclose any significant effects (*all p* > 0.16; *all BFs* < 0.033).

#### TloadDback

Accuracy performance during the 16 min practice on the TloadDback task was computed across four successive 4-min quartiles (t1, t2, t3 and t4; see **Table 2**), and entered in a mixed repeated-measure ANCOVA with within-subject factors Condition (HCL vs. LCL) and Time (t1 vs. t2 vs. t3 vs. t4), between-subjects factor Group (Healthy controls vs. MS patients) and confound covariate Age. The triple interaction Condition × Time × Group did not reach significance (*p* > 0.1, *BF* < 0.33). There was, however, a main Condition effect [*F*_(1,18)__=_ 20.76; *p* <0.001; *MSE* = 0.003; ${\text{η}}_{\text{p}}^{2}$ = 0.54; *BF* > 100] with higher performance in the LCL (95.2 ± 3.5%) than in the HCL (86.7 ± 6.3%) condition. The Condition × Group interaction was also significant [*F*_(1,18)__=_ 8.1; *p* <0.05; *MSE* = 0.003; ${\text{η}}_{\text{p}}^{2}$ = 0.31; BF = 0.64], but Tukey *post hoc* tests failed to disclose between-groups differences in performance either in the LCL (Controls 95.5 ± 3.5% vs. MS 95 ± 3.6%) or the HCL (Controls 88 ± 6.3% vs. MS 85 ± 6.3%) condition (all *p* > 0.6). There was also a significant covariate effect of Age on Condition [*F*_(1,18)__=_ 10.1; *p* <0.01; *MSE* = 0.003; ${\text{η}}_{\text{p}}^{2}$ = 0.36].

**Table 2:** Evolution of accuracy performance across the four quartiles (t1 to t4) during the TloadDback by Condition (HCL vs. LCL) and Group (Controls vs. MS patients).

		TloadDback task		
Group	Condition	t1	t2	t3	t4
Healthy Controls	HCL	90,40 ± 1.9	89,82 ± 2.2	86,56 ± 2.9	85,56 ± 3.3
	LCL	95,96 ± 0.9	96,14 ± 1.3	94,54 ± 1.5	95,24 ± 1.2
MS patients	HCL	84,65 ± 2.3	85,39 ± 1.8	84,65 ± 1.7	86,83 ± 1.8
	LCL	95,86 ± 1.1	94,38 ± 1.6	94,09 ± 1.8	95,57 ± 0.7

*Analyses did not reach significance (p > 0.13; BF < 0.33).*

### fNIRS Results

#### CF-Induction Related COE During Task Practice

As a reminder, COE values were computed across four successive 4-min quartiles (t1, t2, t3, and t4) for each of the 6 target areas (3 per hemisphere), and entered in a repeated-measure ANCOVA with within-subject factors Area (VLPFC vs. DLPFC vs. IPC), Hemisphere (Right vs. Left), Time on Task (ToT; t1 vs. t2 vs. t3 vs. t4) and Condition (HCL vs. LCL), between-subjects factor Group (Healthy controls vs. MS patients), and confound covariate Age. No significant effect was found (*all p* > 0.15).

#### CF-Induction Related COE During Pre- and Post-task Resting States

COE values were similarly recorded for each of the 6 target areas (3 per hemisphere) during the resting states immediately before and after the TloadDback task. A repeated-measure ANCOVA was conducted on COE values during Rst periods with within-subject factors Area (VLPFC vs. DLPFC and IPC), Hemisphere (Right vs. Left), Condition (HCL vs. LCL) and Resting State (pre Rst1 vs. post Rst2), between-subjects factor Group (Controls vs. MS patients) and confounding covariate Age. All effects were non-significant (*all p* > 0.07).

#### CF-Induction Related ALFF During Pre- and Post-task Resting States

The Amplitude of Low Frequency Fluctuations (ALFF) is an index of spontaneous neural activity sensitive to activity changes during resting state sessions (Yu-Feng et al., 2007). A repeated-measure ANCOVA was conducted on ALFF values with within-subject factors Area (VLPFC vs. DLPFC and IPC), Hemisphere (Right vs. Left), Condition (HCL vs. LCL) and Resting State (pre Rst1 vs. post Rst2), between-subjects factor Group (Healthy controls vs. MS patients) and confounding covariate Age highlighted an Area × Group interaction effect [*F*_(2,26)__=_ 4.9; *p* <0.05; *MSE* = 0.003; ${\text{η}}_{\text{p}}^{2}$ = 0.14; BF = 0.27]. Tukey *post hoc* disclosed lower ALFF in IPC than VLPFC and DLPFC both in Controls (*p* < 0.005) and MS patients (*p* < 0.001). Separate ANCOVA in each region of interest failed to disclose significant effects in the VLPFC (all *p* > 0.20), the DLPFC (*all p* > 0.32) and the IPC (all *p* > 0.27).

### Sleep Architecture

To investigate possible structural differences in sleep patterns between patients with MS and Controls (as previously reported, e.g., Côté et al., 2012; Veauthier and Paul, 2014), regular and Bayesian ANCOVAs were computed separately on sleep parameters with Age as a confounding covariate. Given the small size of the sample for these results, relevant values were averaged over the two nights to improve data reliability. No significant difference was found between group but for a higher sleep arousal index in MS patients. Results are reported **Table 3**.

**Table 3:** Polysomnography results.

	Healthy controls (*n* = 10)	Patients with MS (*n* = 9)	*p* (age as covariate)/BF ±
Total Sleep Time (TST; min)	391.7 ± 60.7 ±	380.8 ± 55.5	>0.77/0.43 ±
Sleep Efficiency (TST/SPT)	92.6 ± 6.8 ±	89.4 ± 15.4	>0.91/0.45 ±
Stage N1 (% TST)	6.4 ± 3.6 ±	5.5 ± 2	>0.76/0.56 ±
Stage N2 (% TST)	50 ± 6.1 ±	52 ± 8.5	>0.53/0.46 ±
Stage N3 (% TST)	16.6 ± 5.4 ±	17.3 ± 5.5	>0.36/0.45 ±
Stage REM (% TST)	26.7 ± 9.5 ±	25 ± 4.5	>0.94/0.44 ±
Number of ISA (>2 min)	4 ± 2.3 ±	2.5 ± 1.3	>0.1/1.2 ±
ISA duration (min)	34.7 ± 23.3 ±	37.3 ± 41.1	>0.76/0.41 ±
Arousal index (/h)	2.2 ± 2.3 ±	4.7 ± 3.5	**<0.05^∗^/1.2** ±
Number of Microarousal	15.3 ± 7.6 ±	17.2 ± 4.7	>0.91/0.45 ±
Duration of Microarousal (min)	6.3 ± 0.8 ±	6.6 ± 0.8	>0.54/0.54 ±
Number of Hypoapnea	0.5 ± 1.2 ±	1.3 ± 1.9	>0.76/0.05 ±
Duration of Hypoapnea	3.8 ± 7.16 ±	9.1 ± 11.4	>0.77/0.51 ±
Number of Central Apnea	0.05 ± 0.16 ±	0 ± 0	>0.67/0.55 ±
Duration of Central Apnea	0.9 ± 2.8 ±	0 ± 0	>0.69/0.54 ±
Obstructive Apnea (OSA)	0 ± 0 ±	0.4 ± 0.9	>0.44/0.79 ±
Duration of Obstructive Apnea	0 ± 0 ±	7.6 ± 18.6ˆ*	>0.69/0.62 ±

*Values are mean and standard deviation (m ± std). TST, Total sleep time; SPT, Sleep period time; ISA, Intra sleep awakening. P-values are regular/Bayesian ANCOVAs with age as covariate. ^∗^To note that the duration of OSAs is below 10 s because it represents the average duration among patients; only two of them presented two respiratory events classified as OSA (An apnea must last more than 10 s to be classified as obstructive; Berry et al., 2012).*

### Relationship Between Cognitive Fatigue, Brain Activity and Sleep

Finally, we investigated the potential dual relatioship between the triggering of CF, brain activity patterns and sleep architecture, using three different multiple regression models with between-subjects factor Group (Controls vs. MS patients) and confounding covariate Age. Again, given the restricted sample size, relevant values (self-reported feelings of CF, brain activity and sleep parameters) were averaged over the two sessions to reduce the loss of statistical power due to the inclusion of a new factor in the modeling. Predictors were centered and outliers excluded before computing the analyses.

#### Model A. Relationship Between Brain Activity and Triggering of CF (N Controls = 10; N MS patients = 8)

**FIGURE 4:**
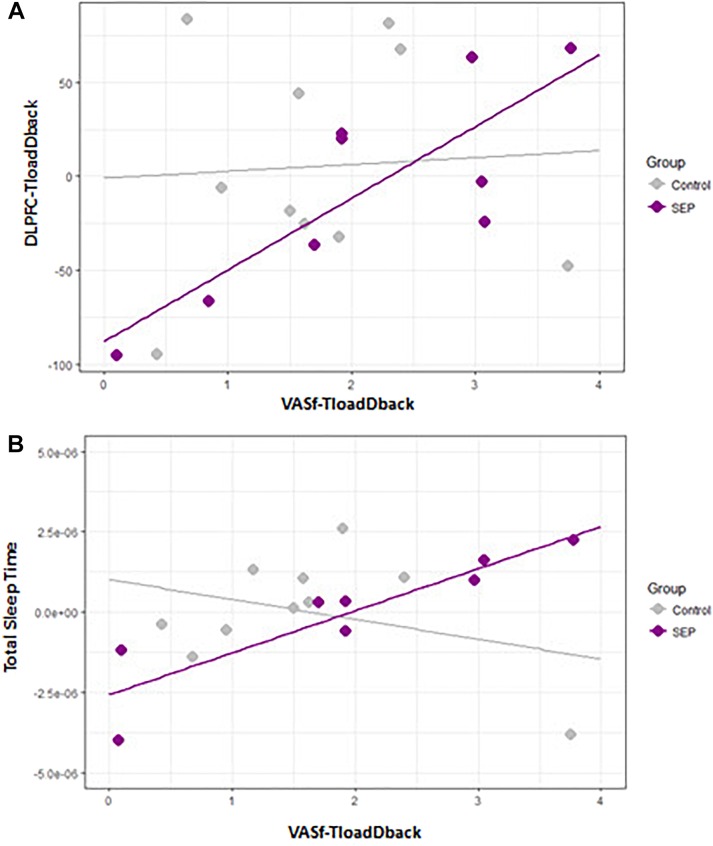
**(A)** Scatterplot illustrating the association between self-reported feelings of cognitive fatigue (CF; VASf) and DLPFC activity (COE). **(B)** Scatterplot between self-reported feelings of CF and total sleep time on the preceding night.

The first model aimed at investigating the relationship between the COE in the three regions of interest (ROIs: VLPFC, DLPFC, IPC) included in the fNIRS set-up (calculated as the average values of COE during TloadDback in both HCL and LCL conditions) and the feeling of CF triggered as a consequence of performing in the TloadDback (i.e., average of self-reported CF during HCL and LCL conditions). COE values at the three ROI were initially included as predictors of CF. Nevertheless, the variance inflation factor (VIF) of the model revealed the existence of collinearity among the predictors (*all VIFs* > 0.7). Therefore, we computed three explorative regression models for each predictor. Models with COE activity in IPC and VLPFC as predictors did not show any significant result (*all p*> 0.1). At variance, COE activity in the DLPFC region exhibited a significant interaction by group (**Figure 4A**), suggesting that activity in this area was associated to the triggering of CF only in MS patients (*p* < 0.005; Adjusted *R* squared > 42% variance). The beta of the slope for the Healthy controls group was -1,7e^05^ whereas for the group of MS patients it was 6.2e^05^.

#### Model B. Relationship Between Sleep Parameters and the Triggering of CF (N Controls = 10; N MS patients = 9)

Given the large number of potential sleep predictors (Côté et al., 2012; # Microarousal, Stages 1-3 and REM, Sleep Efficiency, # ISAs, Total Sleep Time, Arousal index and # of Hypopneas) and the existent collinearity among them, we performed one regression model with each of them. Overall, the predictor Total Sleep Time (**Figure 4B**) disclosed a significant between-group effect (*p* < 0.05; Adjusted *R* squared > 29% variance) suggesting that MS patients who were sleeping more experienced a higher triggering of CF during the TloadDback. Betas were 9.1e-^01^ for the control group and 9.3e-^01^ for the MS control.

#### Model C. Relationship Between Sleep Parameters and CF-Related Brain Activity (N controls = 9; N MS patients = 7)

A new model was created to investigate a possible association between the sleep parameters previously defined and the levels of COE activity in the 3 ROIs. Results failed to disclose significant differences (all *p* > 0.1).

## Discussion

The purpose of the present study was to investigate cortical brain activity and sleep architecture as possible explanatory factors for the triggering of CF in MS disease. To induce comparable levels of CF between healthy controls and MS patients, we used a task that allows adapting cognitive demands to each participant’s optimal performance levels (and cognitive load) during a pretest session (Borragán et al., 2017). Our results show that the manipulation was appropriate for two main reasons. First, there was a significant difference in Pretest STD values between group. Indeed, the final STD was longer at Pretest in patients with MS than in Controls. This suggests that using the same STD for all participants would have resulted from the start in higher cognitive demands in patients with MS than in Controls. This is important because it might have automatically led to higher levels of CF in the patients group. What is more, it may explain why a faster triggering of CF in MS has been reported in prior studies exposing all participants to the same task conditions (Krupp and Elkins, 2000; Lehmann et al., 2012; Claros-Salinas et al., 2013). Second, results of the neuropychological testing showed that patients with MS presented lower scores than Controls on inhibitory and sustained attentional functions as assessed through the Stroop and PASAT tests. Processing speed evaluated via the SDMT and the PASAT was also significantly lower in MS patients. Besides, direct and inverse visual spans scores also suggest that working memory was altered in MS patients, though these latter results were not supported by Bayesian evidence. These results strengthen the need to adapt task demands to the capacities of each individual, especially when the goal of the study is to compare a common phenomenon triggered in two diverse populations. Therefore, the manipulation inherent to the task paradigm used in the present study (TloadDback) helped accounting for cognitive difficulties in determining levels of cognitive load exposure. This fact likely reduced between-group differences in the evolution of self-reported CF, sleepiness and performance in the TloadDback, explaining why we found a similar evolution between the two populations. It can be speculated that equalizing the number of stimuli between groups might have led to a decrease of performance in the MS group, but in this case it would entail that either MS participants would have a faster STD (i.e., being put above their processing capacity) or would need to be exposed longer to the task (being time in itself also a potential contributor in the ToT effect). In this respect, the TloadDback task was designed to allow comparing the evolution of performance across group that exhibit different processing level capabilities. Therefore, task demands (i.e., time needed to process a stimulus) are adapted to each individual eventually leading to similar task durations.

Although the authors acknowledge that a limited sample size in this study asks for caution in the interpretations, Bayesian analyses confirmed an absence of differences in task performance and sleepiness evolution. Moreover, the differential evolution of CF was observed over all the experiment, and was apparently not driven by cognitive load exposure, as patients with MS increased CF within the 5 min of the first resting state session, that is even before the administration of the CF-inducing task. Overall higher fatigability in MS patients was reported in the literature, and might result from the influence of external variables such as anxiety, depression or sleep disorders (lriarte et al., 2000). Notwithstanding those reservations, regression models evidence potential associations between the subjective triggering of CF, brain activity and sleep.

The first regression model disclosed a direct relationship, only present in patients with MS, between brain activity in the DLPFC region and the triggering of subjective CF during the TloadDback task. Diminished oxygenation levels (i.e., higher COE) in the DLPFC were associated with higher feelings of CF. This result may corroborate recent evidence (Sepulcre et al., 2009; Genova et al., 2013) in support of the Chaudhuri and Behan (2000, 2004) model, which proposes that a disruption of the striato-thalamo-cortical pathway is responsible for an exacerbated feeling of CF in neurological disorders such as MS. At variance, a decrease, rather than an increase in activity (oxygenation) associated with higher feelings of CF contrast with the proposal that cerebral overactivation is a causal factor for the triggering of CF in MS (DeLuca et al., 2008; Leocani et al., 2008). Instead, they support earliest evidence suggesting a deactivation in cortical and subcortical regions (Roelcke et al., 1997; Filippi et al., 2002). Another explanation for these different findings relies on task demands (Wylie et al., 2017). Indeed, Wylie et al. (2017) described in healthy participants higher activation levels in the anterior cingulate cortex (ACC) area associated to fatigue when subjects were performing a 2-back task, and deactivation in the same area associated to fatigue during the less complicated zero-back task (Wylie et al., 2017). However, the task condition in our experiment did not appear to modulate changes in cortical oxygenation.

The second regression model evidenced an association between self-reported CF and total sleep time, again only in patients with MS. The analysis suggests that participants who sleep more presented a higher sensitivity to the triggering of CF. That MS patients sleeping longer experience a faster triggering of CF might be explained from two different perspectives. First, it might suggest that patients need longer sleep periods to recover from daytime activity because their sleep is less efficient. Although this possibility was previously discussed (Induruwa et al., 2012; Lehmann et al., 2012), our polysomnography analysis did not support this proposal, considering that patients with MS and Controls exhibited a similar sleep architecture, with only a higher arousal index in patients with MS. Comparable arousal alterations were also reported in MS patients experiencing higher levels of fatigue (Veauthier et al., 2011; Côté et al., 2012). Higher arousal during sleep and increased fatigue might also be associated with a reduced restorative quality of sleep in MS patients. Indeed, sleep in patients with chronic fatigue syndrome was characterized by a disruption in the relative Non-REM sleep power spectra distribution, suggesting a pattern of power exchange in higher frequency bands at the expense of central ultra slow power, that might compromise the homeostatic function of sleep (Neu et al., 2014). Second, it is possible that the higher sensibility of MS patients to the triggering of CF relates to other causes, such as for instance the above-mentioned disruption in the striato-thalamo-cortical pathway. As a consequence, they might require more sleep to recover from the CF accumulated during the day.

Finally, we are willing to acknowledge limitations in our study linked to reduced sample size, and hence reduced statistical power. Nevertheless, the robust nature of our intra-subject comparison models substantiated by Bayesian evidence emphasize the importance of considering individual differences in cognitive capacity prior to the assessment of CF, especially when clinical group are investigated. Besides, mixed models provide further evidence linking cortical brain activity, sleep and CF in MS disease.

## Author Contributions

GB: substantial contributions to acquisition, analysis, interpretation, and drafting and revising of last version to be published. MG: substantial contributions to the analysis of polysomnography recordings. AA: substantial contributions to the statistical analysis. HS: substantial contributions to data acquisition and interpretation of the data. AL: substantial contributions to patient’s recruitment. MDS: substantial contributions to data acquisition. PP: substantial contributions to acquisition, analysis, interpretation, and drafting and revising of last version to be published.

## Conflict of Interest Statement

The authors declare that the research was conducted in the absence of any commercial or financial relationships that could be construed as a potential conflict of interest. The reviewer MMuthalib and handling Editor declared their shared affiliation.
